# The relationship between verb meaning and argument realization: What we learn from the processing of agent-implying intransitive verbs in Japanese

**DOI:** 10.3389/fpsyg.2022.928649

**Published:** 2022-08-01

**Authors:** Zoe Pei-sui Luk

**Affiliations:** Department of Linguistics and Modern Language Studies, The Education University of Hong Kong, Hong Kong, Hong Kong SAR, China

**Keywords:** agent-implying, argument structure, intransitive, Japanese, causal inference

## Abstract

This study investigated whether some Japanese intransitive verbs, called agent-implying intransitive verbs, are processed differently from other ordinary intransitive verbs. These verbs are special in that they denote agentive events, but they are intransitive verbs, which only allow the patient/theme to be the only nominatively marked argument. The priming experiment was designed based on the situation model theory, assuming that verbs with an agentive semantic structure (e.g., ordinary transitive verbs) has a shorter causal inferential distance than those with a non-agentive semantic structure (e.g., ordinary intransitive verb). In the experiment, participants were instructed to read two sentences that formed a story, of which the second sentence was either a transitive or intransitive sentence. The participants then answered a related question about general knowledge, and their response times were measured. The results show that, whereas the mean response time in the ordinary intransitive condition was significantly longer than that in the ordinary transitive condition, the mean response time in the agent-implying intransitive condition was not significantly different from that of the corresponding transitive condition, suggesting that agent-implying intransitive verbs are interpreted as agentive. The findings suggest that agent-implying intransitive verbs instantly evoke agentivity, whereas ordinary intransitive verbs do not. The theoretical implications of the findings are discussed.

## Introduction

Syntactic transitivity has been widely discussed, but there is little behavioral data regarding the processing of verbs with different numbers of arguments. The purpose of this study is to provide such data by testing the online processing of a special type of intransitive verbs in Japanese called agent-implying intransitive verbs (Pardeshi, 2008). By doing so, this study aims at shedding light on the current theories of argument realization.

The article is organized as follows. I first explain the problem presented by some intransitive verbs in Japanese. I then discuss the literature on the relationship between verb meaning and argument realization. Following that will be a description of the method design based on the situation model theory. The results will then be reported, and the interpretations and implications of the results will be discussed.

## Agent-implying intransitive verbs in Japanese

Like many languages, Japanese allows verbs to alternate between transitive and intransitive. For example, the verb that means “open” in Japanese can be realized as an intransitive verb (i.e., *aku* “open”) or a transitive verb (i.e., *akeru* “open”). Japanese, however, is special in that it is rather liberal in allowing a wide range of verbs to enter this transitive/intransitive alternation (Luk, 2014; Matsumoto, 2020). Specifically, it allows some verbs that describe an agentive event to surface as intransitive verbs. For example, the verb *tukamaru* is an intransitive verb to mean “arrest/catch.” The only argument that it accommodates is a patient of the arresting event (i.e., the arrestee). Examples with the transitive and intransitive counterparts are shown in (1a) and (1b), respectively. Note that although the English translation in (1b) is a passive sentence, there is no passive morphology in the original Japanese sentence.


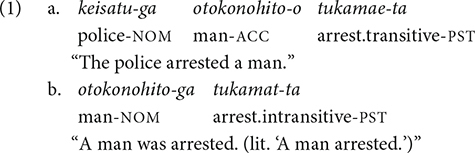


Other examples of this kind of verb include *mitukeru/mitukaru* “find/be found,” *kimeru/kimaru* “decide/be decided,” and *todokeru/todoku* “deliver/be delivered.” The events denoted by all of these verbs require an agent: there must be someone who finds/decides/delivers something. Pardeshi (2008) called these verbs “agent-implying intransitive verbs” (p. 179). Kageyama (1996) pointed out that agent-implying intransitive verbs can be made distinguishable from non-agent-implying intransitive verbs using the phrase *katte-ni* “of one’s own accord,” which is only compatible with the non-agent-implying ones (2).


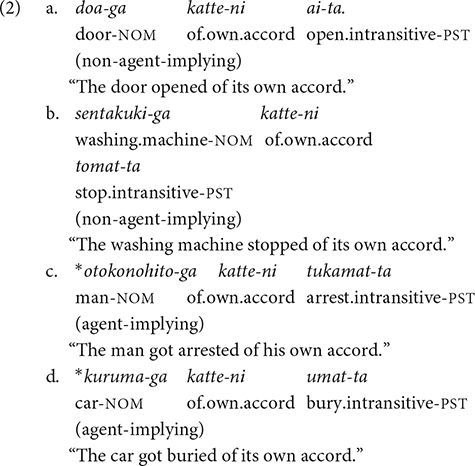


This type of intransitive verbs also occurs in a few South Asian languages, including Hindi, Marathi, Telugu, and Tamil (Pardeshi, 2008). Following Pardeshi (2008), in this article, I use the term ‘‘agent-implying (AI) intransitive verbs’’ to refer to intransitive verbs that denote events that are necessarily agentive^1^, and the transitive counterparts of these intransitive verbs ‘‘AI transitive verbs.’’ Intransitive verbs that are compatible with a non-agentive interpretation are referred to as ‘‘ordinary intransitive verbs,’’ and their transitive counterpart as ‘‘ordinary transitive verbs.’’^2^

It should be noted that AI intransitive verbs is distinct from the middle voice in English, which also involves an agentive verb (e.g., *cut*) without an agent. On the surface, the sentence in (1b) looks like a middle sentence in English, in that it involves a semantically transitive verb appearing with one argument that plays the role of a theme/patient, as in (3). However, it is qualitatively different from an English middle sentence in at least two aspects.


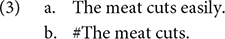


First, an English middle sentence must involve an adverb or an adverbial phrase. The example in (3b) is, therefore, in most cases, unacceptable. Second, semantically, a middle sentence denotes a state instead of an event. The sentence (3a) denotes the property or condition of the meat, rather than a single cutting event (Keyser and Roeper, 1984). In terms of meaning, it is similar to saying *The meat is soft*. These properties distinguish a middle construction from the typical intransitive construction, which usually denotes a single event and does not require any adverbial phrase.

It is also important to note that these AI intransitive verbs are not morphologically derived from the corresponding transitive counterparts. Anticausative, which involves promoting the direct object of a transitive sentence to the subject position with morphological marking, shares some features with the passive, and in some languages the same morphological marking is used in both verbal categories (Kulikov, 1998), but the crucial difference between the two is that, whereas the passive implies the existence of an agent, the anticausative is compatible with an agentless situation that comes about spontaneously (Comrie, 1985). For example, the passive sentence in (4a) is typically interpreted as an event caused by a volitional, animate entity, whereas the intransitive sentence in (4b) can be interpreted as an event brough about by forces in nature, such as the wind. Furthermore, anticausative is often applicable only to verbs denoting events or processes that can be perceived to occur spontaneously (Kulikov, 1998).


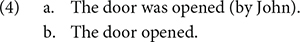


In the case of Japanese, there is no clear anticausative marking on the transitive counterpart of AI verb pairs. For example, the intransitive counterpart *tukamaru* “arrest_intransitive_” even seems to be morphologically simpler than its transitive counterpart *tukamaeru* “arrest_transitive_.” Furthermore, as argued above, it is difficult to perceive the act of arresting to occur without an agent, as shown in the *katte-ni* test in (2). I therefore maintain that AI verbs in Japanese are not cases of morphologically derived anticausatives.

The interesting questions that follow from these AI intransitive verbs are whether they differ from “ordinary,” non-agent-implying intransitive verbs, and if so, how. In the following, I review the literature on the relationship between semantics and syntactic realization, and proceed to explain how the AI verbs in Japanese present a challenging case to our understanding of syntactic realization.

## Lexicalist accounts to argument realization

The start of the discussion of argument realization can be dated back to Chomsky’s Projection Principle and the Theta-Criterion (Chomsky, 1965). In this approach, verbs, as lexical entries, contain information about the number and type of arguments they occur with (i.e., the theta grid). This information is argued to be represented in all syntactic levels (i.e., Logical Form, D-, and S-structure). The Theta-Criterion further specifies that each argument represents one and only one theta-role, and each theta-role is represented by one and only one argument. Theta-roles are involved in the operation of s-selection, which specifies what arguments to be included (Bierwisch, 2006).

Since then, there have been different proposals regarding thematic roles and their mapping to syntax. Baker (1988) proposes the Uniformity of Theta Assignment Hypothesis, which states that identical thematic relationships are mapped onto identical structural relationship at the D-structure. Specifically, an Agent is uniformly mapped onto the Spec of a VP at D-structure, which subsequently moves to the subject position at S-structure. Fillmore (1968) proposes a thematic hierarchy such that an agent is ranked higher than an instrument, which in turn is ranked higher than a patient or theme in terms of their priority of subject selection. In other words, if there is an agent, it becomes the subject of the sentence. Dowty (1991) argues that thematic roles should be understood in terms of agent proto-roles and patient roles, with each of them having a list of properties (e.g., volitionality). In his approach, the argument that has more properties of the agent proto-roles becomes the subject. The assumption common to these approaches is that an agent, if it exists in an event, is a core participant. They also come to the same conclusion that if there is an agent or external causer, it will be placed in the Subject position.

Some researchers argue that whether an agent is a necessary argument largely depends on the semantics of the verb. Levin (1993) and Guerssel et al. (1985) argue that *cut* cannot be intransitive because of its semantic properties. They showed that the verbs that correspond to “cut” in four typologically different languages only occur as a transitive verb. Levin (1993) explained that the verb *cut* can never be intransitive, because the act of cutting involves an instrument and an intentional agent who uses the instrument. Therefore, *cut* can never appear as an inchoative (intransitive) verb. Levin and Rappaport Hovav (1995) further argue that causative alternation is only possible for predicates that allow both agent and causer (inanimate entities) to function as external arguments. Haspelmath (1993) also argues that “the most important specific semantic condition on inchoative/causative verb pairs is the absence of agent-oriented meaning components” (p. 93). He contrasted *cut* with *tear*, where *cut* has the agent-oriented component, because it involves the use of a sharp instrument, whereas *tear* does not, allowing *tear* to appear as an intransitive verb. Pinker (1989) also holds that the semantics of a verb determines its syntactic behaviors. He argues that only transitive verbs that denote events that are [-contact] and [-motion] can enter the anticausative alternation. This, according to Pinker, explains why *break* has an intransitive counterpart, whereas *hit* and *cut* do not. In sum, these semantic accounts argue that the syntactic behaviors of verbs are largely determined by the semantics of the verbs. In particular, Levin (1993) and Haspelmath (1993) contend that a verb that denote a necessarily agentive causative event cannot be intransitive.

Researchers such as Pustejovsky (1991) and Rappaport Hovav and Levin (1998) systematically describe the relationship between argument structure and verb semantics in the form of event structure. Following Vendler’s four-way predicate classification, Rappaport Hovav and Levin (1998) argue that lexical aspect systematically varies with event structure. For example, manner verbs such as *sweep* are activities and have the event structure in (5), whereas result verbs are either achievements or accomplishments, having the event structures in (6) and (7), respectively, where X and Y are participants in the event.


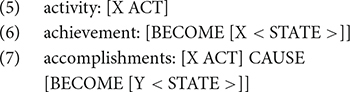


In their approach, there are two types of participants, structure and content. Structure participants are those licensed by the event structure template shared by other members of the same verb class, whereas content participants are those that are licensed by the meaning of the verb. For example, the verb *sweep* is an activity having an event structure template in (5). The template licenses an agent, making the agent a structure participant. The verb also allows a constant participant, which is a surface on which the sweeping act occurs, such as *the floor*. This is licensed by the semantics of the verb: sweeping must involve a surface.

To explain how arguments are realized based on event structure templates, Rappaport Hovav and Levin (1998) posit two well-formedness conditions. First, the Subevent Identification Condition states that “(e)ach subevent in the event structure must be identified by a lexical head (e.g., a V, an A, or a P) in the syntax” (p. 16). Second, the Argument Realization Condition (ARC) states that “(t)here must be an argument XP in the syntax for each structure participant in the event structure” and “(e)ach argument XP in the syntax must be associated with an identified subevent in the event structure” (p. 17). For example, the verb *sweep*, when denoting an accomplishment, includes two subevents, as shown in (8), which are associated with two structure participants, “Phil” and “dust.” According to the ARC, both roles should appear in the syntax. The sentence in (9) is therefore ungrammatical because the structure participant from the second subevent (i.e., “dust”) is missing.


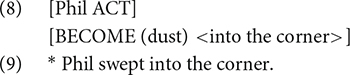


Acknowledging that many verbs, especially manner verbs, can have more than one syntactic realization, Rappaport Hovav and Levin (1998) argue that a semantic structure can be augmented to include other subevents. For example, the verb *sweep*, which is typically a manner verb, can be augmented from an activity to an accomplishment, as in (8). To account for the fact that *sweep* can also be used as an intransitive verb, Rappaport Hovav and Levin argue that the constant participant of *sweep* (i.e., a surface on which the act of sweeping occurs) is optional, because it is prototypically “a floor” (p. 19) and is recoverable. In sum, Rappaport Hovav and Levin maintain that syntactic realization is a direct projection of the event structure of a verb.

Based on Levin and Rappaport’s analysis, one might predict that verbs that denote agentive causative events will not be lexicalized as intransitive verbs, because an agent is a structure participant, and it must be realized in the syntax, unless the verb is morphologically marked (e.g., in the form of the reflexive morpheme in Spanish), as they argue. In English, for example, a causative event with the agent backgrounded requires a passive construction (10a). As discussed in many works (e.g., Roeper, 1987; Alexiadou et al., 2008), the difference in causality between a passive construction and an intransitive construction can be shown by the fact that one can add a *by*-phrase to the passive sentence to indicate the agent or causer, as in (10a), but not to an intransitive sentence, as shown in (10b).


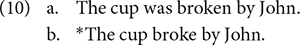


The ARC thus nicely explains the absence of *cut* as an intransitive verb. Levin (1993) claims that the act of cutting involves the use of an instrument, which has to be manipulated by an agent. In terms of the ARC, since one of the subevent involves an actor/agent, this argument has to appear in the syntax, and thus using *cut* as an intransitive verb is not an option.

Pinker (1989) proposes the Grammatically Relevant Subsystem hypothesis, which claims that linguistic processes are only sensitive to a subset of semantic elements that human beings are able to perceive. For example, causation is systematically encoded in languages, but not the physical setting of an event (e.g., temperature). Following Jackendoff’s Lexical Conceptual Structure, Pinker argues that these selected semantic elements are systematically reflected in syntax. In the case of an act involving two participants, the agent is linked to the external argument, and the patient is linked to the direct internal argument. His analysis would imply that, if an act is understood to be caused by an agent, the verb that denotes that act will include an agent in the semantic structure, which will be represented in the surface form of the sentence as an external argument through the relevant linking rule.

There, however, does not seem to be a satisfactory explanation as to how the number and type of arguments are determined. Rappaport Hovav and Levin (1998) argue that the constant participants are determined by the semantics of a verb: the act of running minimally involves a runner and the act of sweeping minimally requires a sweeper and a surface. However, one might also argue that the act of running requires a surface to run on, or the act of sweeping requires a tool. Pinker also does not explain what will be included in the semantic structure in the first place. In his explanation, he took it as a matter fact that *kiss* is a transitive verb (i.e., should take two arguments) because the act involves two participants.

In fact, there exist examples that contradict the ARC. Otsuka’s (2007) informant found (11) acceptable, although it does not conform to the proposal of Rappaport Hovav and Levin (1998) in that the sentence only allows part of a subevent (i.e., the theme) to appear in the sentence while omitting the state of the theme (e.g., *into the corner*).


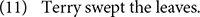


Another example is the Japanese verb *kiru* “cut.” Contrasting with Levin’s (1993) claim, *kiru*, the transitive verb in Japanese that corresponds to *cut* in English, has an intransitive counterpart *kireru*. In addition to a non-agentive interpretation, in which case the verb means “to break apart,” the verb can be used in a situation where an instrument is clearly involved in an event, as shown in (12).


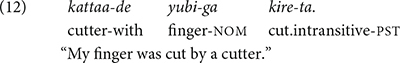


If the syntactic structure of a verb is solely determined by its event structure, then we are obliged to interpret the AI intransitive verbs as verbs that denote only the end-state and no action is involved. That is, *tukamaru* in (1b) only denotes a state in which the man is in captive, and no act of arresting is involved in the event structure of *tukamaru*. But if *tukamaru* does involve an agent as part of its lexical meaning, then it may suggest that the syntactic structure cannot be directly inferred from the event structure of a verb, and alternative views to argument structure are called for.

## Alternative views to argument structure

Jackendoff (1990) offers a different view. He proposes that each lexical item has a lexical conceptual structure, which is part of the lexical entry and describes the mental representation of the lexical item. This lexical conceptual structure, however, does not directly linked to the syntactic structure. The syntactic structure is a separate layer that a lexical entry needs to specify, corresponding to the syntactic structure through coindexing. In other words, in Jackendoff’s approach, not all entities in the conceptual structure need to be expressed in the syntactic structure. For example, (13) shows the lexical entry of the verb *open*. The entry lists the part of speech and conceptual structure of *open*. Although the event denoted by the verb is conceptually causative, as shown by the conceptual category CAUSE, the agent is optional, indicated by the underline.


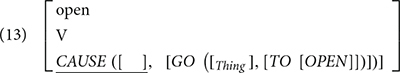


Many authors argue that some of the arguments are in fact introduced *via* syntax, and this has become the main approach in the recent generative work (see Marantz, 2013 for a summary). For example, Kratzer (1996) and Alexiadou et al. (2015) argue that the external argument of an event is base-generated in the Spec of the Voice Phrase. In other words, the external argument (i.e., the agent) is not projected through the theta-grid of a verb, but is added through syntax (Alexiadou et al., 2015). Alexiadou et al. argue that both causative and anticausative verbs include a cause and thus do not differ in event complexity. The external argument is introduced *via* a Voice Phrase. This is illustrated in (14) and (15) (adapted from Alexiadou et al., 2015).


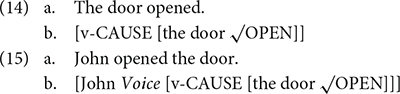


Construction Grammar is even more extreme as to how much is attributed to syntax/constructions. Unlike the claim that the syntactic structure is a projection of the properties of the verb in a sentence, the constructional view holds that argument structure is largely determined by the construction that the verb occurs in Goldberg (2010, 2005), Goldberg and Jackendoff (2004); Goldberg (1995). For example, the verb *sneeze*, which is often understood to be intransitive, can occur in a Cause-Motion Construction (16), which has three arguments. In other words, the constructional view argue that even non-external arguments are introduced at a syntax/construction level.


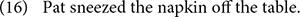


## Summary of theories of argument realization

The discussion regarding argument structure mainly concerns how much information regarding argument realization is specified in the lexical entry of a verb and how much comes from syntax. At one end of the debate, the semantics of verb are argued to have direct syntactic consequences; at the other end, argument configuration is partly or largely determined by syntax or construction. These discussions, however, mainly rely on examples in languages (mostly English), and there is little behavioral data regarding these positions. The current study fills this gap by investigating whether AI intransitive verbs in Japanese are processed differently from ordinary intransitive verbs by native Japanese speakers. If the AI intransitive verbs are shown to be processed similarly to ordinary intransitive verbs, it suggests that the two kinds of verbs in nature and the processes involved in their interpretations. However, if they are shown to be processed differently, it may suggest that the two kinds of verbs have different semantic or event structures, or the processes involved in their interpretations are different. The findings will have implications on each of these theories.

## The study

To investigate whether ordinary and AI intransitive verbs are processed differently, an experiment designed based on the situation model theory was used. This section gives a brief description of the situation model and explains how it can be used to address the research question.

### The situation model

According to the situation model theory, when a reader reads a narrative, he/she constructs a mental world with his/her linguistic, pragmatic, and world knowledge (e.g., Kintsch and van Dijk, 1978; Magliano and Schleich, 2000). The theory has been tested in different domains, including temporal (e.g., Rinck et al., 2001; Therriault and Raney, 2007), spatial (e.g., Zwaan and van Oostendorp, 1993; Hakala, 1999; Blanc and Tapiero, 2001; Dutke et al., 2003), causal (e.g., Trabasso and van den Broek, 1985; Fletcher and Bloom, 1988; Singer et al., 1992; Suh and Trabasso, 1993; Blanc et al., 2008), person- and object-related information (e.g., Wilson et al., 1993; de Vega, 1995; Radvansky et al., 1997).

To construct a situation model, a reader must first read a sentence, which is referred to as the surface code. The reader then derives a textbase, which is a text proposition, based on the words and syntax of the surface code. Finally, the reader combines the textbase with world knowledge and experience to create a situation model.

A few studies investigated how linguistic forms affect the construction of situation models. For example, Magliano and Schleich (2000) tested how aspect markers affect the activation of an event during the construction of a situation model. In one experiment, the participants were asked to read passages that contained either a sentence with an imperfectively marked verb (e.g., *Betty was delivering their first child*) or one with a perfectively marked verb (e.g., *Betty delivered their first child*). The participants continued with the passage until a verb phrase appeared on the screen, and the participants had to indicate as quickly and accurately as possible whether the verb phrase occurred in the passage. They found that the participants responded faster after reading a sentence with an imperfectively marked verb than after reading one with a perfectively marked verb. They argue that activation for in-progress actions is maintained longer than completed actions, allowing them to respond faster in the former condition. They thus argue that grammatical markers contribute to the construction of situation models.

Singer et al. (1992) were interested in how people make causal inferences. In the first of a series of three experiments, they tested whether causal inference was made when situation models were constructed. The participants read two-sentence passages with either (17a) or (17b), and responded to a question regarding general knowledge as in (17c):


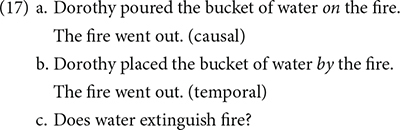


They found that participants who read (17a) responded significantly faster to the question (17c) than those who read (17b). They explained that this was because the participants who read the (17a) constructed a situation model in which the water extinguished the fire as a result of Dorothy pouring water on it. The participants were therefore primed by the causal situation model constructed when they had read (17a), but no such priming happened when they had read (17b), because the situation model constructed was not a causal one.

The third experiment of their study tested the effect of inferential distance on the construction of a situation model. In this experiment, there were the near causal (18a), the far causal (18b), and the temporal (non-causal) (18c) conditions. The participants read one of these sentences, and then continued to read (18d), and responded to the question (18e).


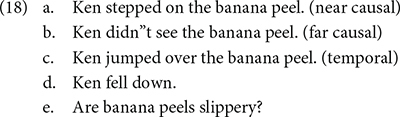


They found that the participants in the near causal condition responded to the question (18e) significantly faster than the temporal one, and marginally significantly faster than those in the far causal condition.

Adopting Singer et al.’s methodology, the current study used a priming experiment that tests causal inferential distance by manipulating the linguistic form (i.e., transitive vs. intransitive forms). Transitivity is closely related to causality. The use of the transitive construction is an indication of a direct/near causal event (i.e., a causal inference is made using linguistic devices). For example, (19a) indicates that John directly caused the window to break. On the contrary, the use of the intransitive construction denotes a far causal event (i.e., a causal inference is indirectly made through world knowledge) or simply a sequence of events (i.e., no causal inference is made). For example, (19b) involves no indication of direct causation. If it is followed by a sentence such as *John shot at the intruder*, the causation can be inferred (far causal) or the two events can have no cause-and-effect relationship (temporal).


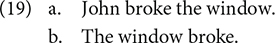


Based on the findings of Singer et al. (1992), it was hypothesized that items with an ordinary transitive verb should produce shorter response times than those with an ordinary intransitive verb if a similar methodology is adopted. With these assumptions, the study investigated whether AI intransitive verbs are interpreted to be causative. The study hypothesized the following: if AI intransitive verbs are interpreted to be causative, the reaction times to items involving these verbs should not be significantly different from their transitive counterparts^3^; on the other hand, if they are not interpreted to be causative, the reaction times to items involving these verbs should be significantly longer than their transitive counterparts.

## Method

Before administering the priming experiment, a norming task was conducted to ensure the validity of the test items.

### Norming task

Whether a verb is agent-implying or not could vary with the context due to slightly different senses of a verb. For example, *tuku* can mean “attach,” which may not require any agent, but when the predicate is *denki ga tuku* “the light is switched on,” the switching on of the light almost always involves an agent. The purpose of the norming task is therefore to test whether the context created in the test items would normally be understood to involve an agent.

#### Participants

Twenty adult native Japanese speakers participated in the norming task. Seventeen of them resided in Japan and three in the United States when the task was administered. No compensation was offered to the participants.

#### Materials

To construct the test items, 40 verb pairs, 20 ordinary pairs and 20 agent-implying pairs, were selected from a comprehensive list of Japanese verbs in Jacobsen (1991). The ordinary verb pairs involve an intransitive counterpart which typically describes events that can happen spontaneously. For instance, the act of melting can be easily seen as a natural process without an intentional agent (e.g., the ice-cream melted). Therefore, *tokeru/tokasu* “melt_*intransitive*_/melt_*transitive*_” were used to construct the ordinary items. These ordinary intransitive verbs in Japanese have a corresponding intransitive verb in English, which are argued to be agentless (Haspelmath, 1993; Levin, 1993). The AI verb pairs, on the other hand, involve an intransitive counterpart that typically describes an event that does not happen spontaneously without an agent. A typical example is *tukamaru* “catch/arrest. intransitive”/*tukamaeru* “catch/arrest.transitive. These AI verbs pairs are, on the other hand, verb pairs whose intransitive counterparts are not lexicalized in English. Most of these verbs were translated as passive in English in Jacobsen’s (1991; see Table 1).

**TABLE 1:** Japanese verbs used in the norming task and the priming experiment.

**Ordinary transitive**	**Ordinary intransitive**	**AI transitive**	**AI intransitive**
*kowasu* “break”	*kowareru* “break”	*mitukeru* “find”	*mitukaru* “be found”
*okosu* “get up”	*okiru* “get up”	*tukamaeru* “arrest”	*tukamaru*“be caught”
*narasu* “ring”	*naru* “ring”	*tasukeru* “help”	*tasukaru* “be helped”
*kudaku* “shatter”	*kudakeru* “shatter”	*turu* “fish”	*tureru* “be caught (of fish)”
*tomeru* “stop”	*tomaru* “stop”	*soroeru* “collect”	*sorou* “be collected”
*hiyasu* “cool”	*hieru* “cool”	*sadameru* “decide”	*sadamaru* “become decided”
*kogasu* “burn” “scorch”	*kogeru* “burn” “become scorched”	*sonaeru* “provide with”	*sonawaru* “be provided”
*kobosu* “spill”	*koboreru* “spill”	*tunagu* “connect”	*tunagaru* “become connected”
*ugokasu* “move”	*ugoku* “move”	*kimeru* “decide”	*kimaru* “become decided”
*korogasu* “roll”	*korogaru* “roll”	*mazeru* “mix”	*mazaru* “become mixed”
*taosu* “collapse”	*taoreru* “collapse”	*ueru* “plant”	*uwaru* “be planted”
*mawasu* “turn”	*mawaru* “turn”	*tirakasu* “scatter”	*tirakaru* “become scattered”
*nejiru* “twist”	*nejireru* “twist” “become twisted”	*tutaeru* “transmit”	*tutawaru* “be handed down” “be transmitted”
*fuyasu* “increase”	*fueru* “increase”	*todokeru* “deliver”	*todoku* “be delivered” “arrive”
*tokasu* “melt”	*tokeru* “melt”	*umeru* “bury”	*umaru* “be buried”
*akeru* “open”	*aku* “open”	*someru* “dye”	*somaru* “be dyed”
*waru* “break”	*wareru* “break”	*hameru* “fit (something) in”	*hamaru* “fit in”
*muku* “peel”	*mukeru* “peel”	*tukeru* “switch on”	*tuku* “be switched on”
*sodateru* “bring up”	*sodatu* “grow up”	*nuku* “pull out”	*nukeru* “come out”
*katamukeru* “tilt”	*katamuku* “tilt”	*tateru* “build”	*tatu* “be built”

Eighty items were constructed using the ordinary verbs and 40 using the AI ones. The same items were used in the priming experiment reported below. The 120 items were equally divided into two versions, such that a participant would only see the transitive or intransitive version for a given context. Three pairs of AI verb items and four pairs of ordinary verb items were later removed for analysis because they did not pass the *katte-ni* test shown in (2).^4^

#### Procedure

The task was a paper-based task with Japanese orthography (i.e., Hiragana, Katakana, and Kanji). All participants completed that task in a classroom on the campus of the university where they were recruited. An example of an item is shown in (20a) and (20b)/(20c). Each item consisted of three sentences. The first sentence (20a) introduces a context. The second sentence is either an intransitive sentence (20b) or a transitive sentence (20c). They read the sentence pairs and indicated how likely the event in (17b) was caused by the person mentioned in (20a) by rating a statement like (20d) on a 7-point scale, 7 being very likely and 1 being very unlikely. Each participant took 10–15 min to complete the task.


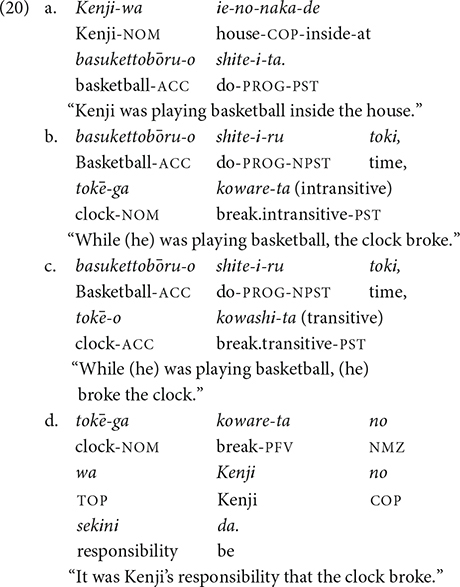


#### Results

**FIGURE 1:**
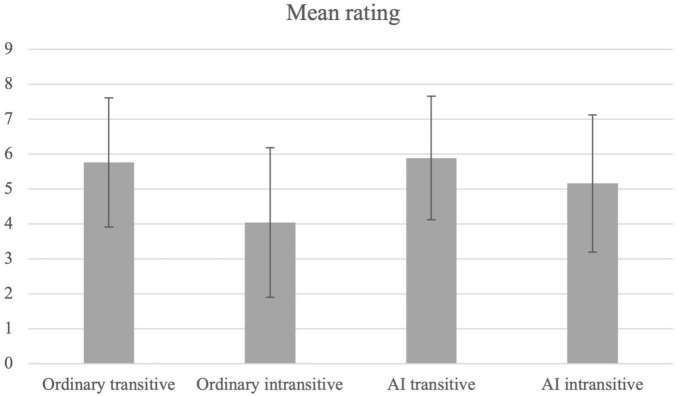
Mean ratings of causality (error bar = 1 SD).

The mean ratings for the different constructions and their standard deviations are summarized in Table 2 and Figure 1. The data were first transformed using a Box-Cox transformation. The transformed data were then fitted to a generalized linear mixed model using glmer() in the lme4 package for R (Bates et al., 2015) with the construction type as the fixed variable and participants and items as the random variables. The model was significantly different from the null model with the random variables only [χ^2^(3) = 181.55, *p* < 0.001], revealing a main effect for construction type. Pairwise comparisons were performed using glht()in the multcomp package (Hothorn et al., 2008). As expected, a significant difference was found between the ordinary transitive and ordinary intransitive conditions, with the ordinary transitive rated higher than the ordinary intransitive condition (*z* = 13.124, *p* < 0.001). Interestingly, the mean rating of the AI intransitive items was significantly greater than that of the ordinary intransitive items (*z* = −4.748, *p* < 0.001), indicating the AI intransitive items were more likely to be interpreted as agentive than the ordinary intransitive items. Significant differences were also found between AI intransitive and the AI transitive conditions (AI intransitive < AI transitive, *z* = 4.032, *p* < 0.001), between AI intransitive and the ordinary transitive conditions (AI intransitive < ordinary transitive, *z* = 2.599, *p* < 0.05), and between the ordinary intransitive and the AI transitive conditions (ordinary intransitive < AI transitive, *z* = −7.983, *p* < 0.001). No significant difference was found between the ordinary transitive and the AI transitive conditions (*z* = −0.633, *p* = 0.917). The statistical model and the pairwise comparisons are shown in Tables 3, 4 respectively.

**TABLE 2:** Mean ratings of causality and standard deviations.

	Ordinary transitive	Ordinary intransitive	AI transitive	AI intransitive
Mean	5.76	4.04	5.89	5.16
SD	1.85	2.14	1.77	1.96

**TABLE 3:** Generalized linear mixed model for the rating data.

Fixed effects:			
	**Estimate**	**SE**	***t*-Value**
(Intercept)	7.620	0.479	15.906
AI transitive	1.599	0.397	4.032
Ordinary intransitive	–2.347	0.494	–4.748
Ordinary transitive	1.286	0.495	2.599

AI intransitive is the reference.

**TABLE 4:** Pairwise comparisons of ratings.

	Estimate	SE	*z*-Value	Pr(>| *z*|)
A_transitive – A_intransitive = 0	1.599	0.397	4.032	<0.001 ***
Intransitive – A_intransitive = 0	–2.347	0.494	–4.748	<0.001 ***
Transitive – A_intransitive = 0	1.286	0.495	2.599	0.043*
Intransitive – A_transitive = 0	–3.945	0.494	–7.983	<0.001 ***
Transitive – A_transitive = 0	–0.313	0.495	–0.633	0.917
Transitive – intransitive = 0	3.632	0.277	13.124	<0.001 ***

A_transitive = AI transitive, A_intransitive = AI intransitive, intransitive = ordinary intransitive, transitive = ordinary transitive. ***p < 0.001, *p < 0.05.

#### Discussion

The participants rated the items higher in the ordinary transitive condition than in the ordinary intransitive condition. They also rated the AI intransitive verbs significantly higher than the ordinary intransitive verbs. The results suggest that the items in the AI intransitive condition are more likely to be understood to involve an agent, whereas the ordinary intransitive verbs are neutral about the involvement of agent. The significant difference between the AI transitive and the AI intransitive conditions was unexpected, as we expected that both types of verbs are agentive. It might be because the statements for rating specify the agent, as in “it was Kenji who is responsible for….” Since the AI intransitive items did not specify who caused an event to happen, the participants might interpret the event to be caused by someone else, someone not mentioned in the discourse. Thus, the unexpected difference found might be because the participants allowed an interpretation that the event was caused by someone different from the person mentioned.

### The priming experiment

The question of interest is whether AI intransitive verbs are different from ordinary intransitive verbs in online processing, which can inform us about the difference in nature between these two types of verbs. A priming experiment was conducted to address this question.

#### Participants

A different group of 46 native Japanese speakers participated in the experiment. All of them were undergraduate students at a university in Japan. They were compensated with JPY¥2000 (about USD$16) for their participation.

#### Materials

The priming experiment was designed based on Singer et al. (1992). Unlike Singer et al. (1992), however, the experiment manipulated the linguistic information to test whether causality was evoked with different verb types. The materials used the same sentences as those used in the norming task. The difference is that, instead of eliciting offline judgments regarding whether the event was caused by the person mentioned in the sentence pairs, they were asked to respond to a question about world knowledge, which asked whether an object mentioned in the first sentence can act as an instrument or means for the event described in the second sentence. An example is shown in (21).


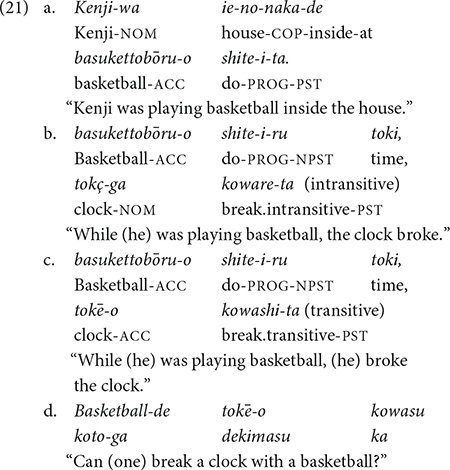


The rationale of the experiment is that the use of transitive verbs in a sentence resembles the near causal condition in Singer et al. (1992), as a transitive verb (21c) involves an agent, and thus the participants would have constructed a causative situation model with the use of the object mentioned in the first sentence as an instrument, which should activate the general knowledge involved in the general knowledge question (21d) and thus shorten their response times to the question. The use of the intransitive verb in a sentence, on the other hand, resembles a far causal or temporal conditions in Singer et al. (1992). For example, the breaking of the clock in (21b) may not be caused by anyone, and so the relationship between the two events in (21b) can be purely temporal. Participants, therefore, should have a longer response time to the question, because there is a longer causal inferential distance (Singer et al., 1992).

All participants read (21a), and then they read either the intransitive version (21b) or the transitive version (21c). After reading the two sentences, they were asked to respond to the question (21d) as quickly and as accurately as possible. The question (21d) was followed by a comprehension checking question “did John play inside the house?” This was to ensure that participants actually read the sentence pairs during the task.

Because the frequency of the verbs may have an influence on the processing of the sentences and the response times of the participants, the frequencies of these verbs were looked up in *Nihongo No Goi Tokusei* (Lexical properties of Japanese) (Amano and Kondo, 2000). It is a database that lists the frequencies of the words used in news articles published from 1985 to 1998. To ensure that the frequencies of the verbs in different conditions are comparable, an ANOVA was run to compare the frequencies of occurrence of the verbs among different conditions. Results from the ANOVA show that the four types of verb were not significantly different in terms of frequency of occurrence [*F*(3,76) = 0.782, *p* = 0.507].

Apart from the target items in the four different conditions (ordinary transitive, ordinary intransitive, AI transitive, and AI intransitive), there were two other types of items. The filler items included a general knowledge question about an object mentioned in the first sentence, but is not related to the situation. For example, after reading *John was watching a movie with a bowl of popcorn. While he was watching, the popcorn spilled*, the participants had to answer the question *Is popcorn made from plastic?* The purpose of these items is to elicit “no” as the answer for the first question. There were 40 of them. The second type, the baseline items have a similar pattern to the target items, but the second sentence is purely a temporal one. An example would be *Mary was washing dishes. While she was rinsing the dishes, the phone rang*, and the participants had to answer the question *Are dishes breakable?* There were 20 baseline items. The 120 target items were divided equally in two versions such that a participant would not respond to both conditions for a given context. The same fillers and baselines questions appear in both versions.

#### Procedures

The experiment was conducted using the software E-prime 2.0, and the participants read the sentences and questions from a computer screen. The participant read in a self-paced manner. The participants first read the first sentence. When they had understood the sentence, they pressed the spacebar to continue. Then the second sentence appeared. After they had understood the sentence, they pressed the spacebar again. A fixation “+” then appeared for 500 ms. Then the first question (i.e., the general knowledge question) appeared. The participant had to answer as quickly and accurately as possible to the question within 5 s. If no response was received in 5 s, the answer was considered as “incorrect.” After the participants had answered the first question, the second question appeared. The participants again had to answer the question in 5 s. After the question was answered, the next item appeared, and the procedures repeated.

Eight training items unrelated to the purpose of the present study were administered to the participants in the beginning of the task to familiarize them with the procedures. All items in the trial session were randomized in such a way that all participants did the items in a different order.

#### Results

**FIGURE 2:**
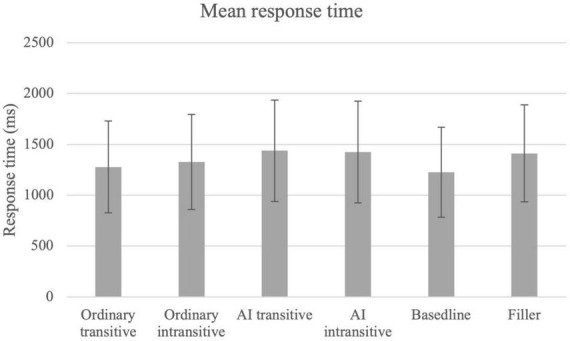
Mean response times (error bar = 1 SD).

The items that did not pass the *katte-ni* test (i.e., three pairs of agent-implying verb items and four pairs of non-agent-implying verb items) were excluded for analysis. In addition, only the items of which the responses to both questions were correct were included for analysis (85.7%). Long response times, whose natural logarithms were larger than eight,^5^ were further excluded (2.6%). The logarithms of the response times to the first question were fitted to a linear mixed-effect model using lmer() in the R package lme4 (Bates et al., 2015), with construction types as the fixed effect, and Subject, Trial, Item (verb), and the number of characters (hiragana, katakana, and Kanji) in the target question as random effects. A null model was also fitted with only the random factors. Results show that the model with construction type as the fixed effect is significantly different from the null model [χ^2^(5) = −163.80, *p* < 0.001], suggesting that the type of construction is a factor affecting the response times of the participants. *Post hoc* Tukey pairwise comparisons using glht()in the R package multcomp (Hothorn et al., 2008) revealed a significant difference between ordinary transitive and ordinary intransitive (ordinary transitive < ordinary intransitive, *z* = −2.877, *p* < 0.05), but there was no significant difference between the AI transitive and the AI intransitive conditions. Significant differences were also found between ordinary transitive and AI transitive (ordinary transitive < AI transitive, *z* = −3.133, *p* < 0.05), as well as between ordinary transitive and AI intransitive (ordinary transitive < AI intransitive, *z* = −3.223, *p* < 0.05). The mean response times are summarized in Table 5 and Figure 2, and the statistical model and the pairwise comparisons are shown in Tables 6, 7 respectively.

**TABLE 5:** Mean response times and standard deviations.

Construction	Ordinary transitive	Ordinary intransitive	AI transitive	AI intransitive	Baseline	Filler
Response times (ms)	1,278	1,328	1,438	1,426	1,226	1,412
SD	453	469	499	501	444	478

**TABLE 6:** Fitted mixed-effect generalized linear model for the response times.

	Estimate	SE	t-Value
(Intercept)	7.198	0.053	136.343
AI transitive	–0.004	0.018	–0.226
Baseline	–0.079	0.055	–1.436
Filler	0.035	0.047	0.744
Ordinary intransitive	–0.111	0.046	–2.435
Ordinary transitive	–0.148	0.046	–3.223

AI intransitive is the reference.

**TABLE 7:** Pairwise comparison of response times.

	Estimate	SE	*z*-Value	Pr(>| *z*|)
A_transitive – A_intransitive = 0	–0.004	0.018	–0.226	1.000
Intransitive – A_intransitive = 0	–0.111	0.047	–2.435	0.116
Transitive – A_intransitive = 0	–0.148	0.046	–3.223	0.012 *
Intransitive – A_transitive = 0	–0.107	0.046	–2.345	0.143
Transitive – A_transitive = 0	–0.143	0.048	–3.133	0.016*
Transitive – intransitive = 0	–0.04	0.013	–2.877	0.036 *

A_transitive = AI transitive, A_intransitive = AI intransitive, intransitive = ordinary intransitive, transitive = ordinary transitive. Comparisons involving baseline and filler items are not reported here for clarity reasons. *p < 0.05.

#### Discussion

The results of the priming experiment show that the participants responded to the items in the ordinary transitive condition significantly faster than to those in the ordinary intransitive condition, confirming the prediction that the ordinary transitive verbs produce a smaller inferential distance than the ordinary intransitive verbs. We assume that this smaller inferential distance was due to the direct causation expressed by the transitive verbs, which is absent in the intransitive verbs based on the rationale of Singer et al. (1992), the ordinary transitive verb triggers the construction of a causative situation model, which involves the act of the agent, and activates the concepts involved in the general knowledge question. The ordinary intransitive condition, on the other hand, does not trigger the construction of a causative event, and thus no general knowledge involved in the question was activated before the general knowledge question was encountered. Therefore, the transitive condition has a shorter mean response time than the intransitive condition.

However, the mean response times of the AI transitive and intransitive verb pairs were not significantly different, suggesting that there is no or little difference in inferential distance. Since AI transitive verbs denote agentive events, the lack of significant difference between the agent-implying transitive and intransitive verb pairs would suggest that the AI intransitive verbs are also understood to be causative, even though the AI intransitive verbs do not have a visible agent. In other words, we can infer that the general knowledge involved in the general knowledge question was activated before the general knowledge question when the participants read the sentences with AI intransitive verbs. Although it is not clear whether the activation of general knowledge is semantic or pragmatic in nature, there is one thing we can conclude: whereas the sentences in the ordinary intransitive condition do not instantly activate the causation-related general knowledge, sentences in the AI intransitive condition do.

What is unexpected was the difference between the ordinary transitive and AI transitive conditions, and the significantly longer response times for the two AI conditions. I speculate that this might be due to the non-prototypical nature of AI transitive verbs. Prototypical transitive verbs denote causative events in which an entity undergoes internal changes (Hopper and Thompson, 1980; Tsunoda, 1985). AI transitive verbs often only indicate change in location (Luk, 2014). At the same time, the AI intransitive verbs are also atypical in that they are agentive with an invisible agent. Research has shown that typical items in a category are usually processed faster than atypical ones (e.g., McCloskey and Glucksberg, 1978; Larochelle and Pineau, 1994). The longer response times of the AI conditions might be due to their non-prototypicality. However, this remains a speculation, and I leave this to future research.

## General discussion

As predicted, the ordinary intransitive items produced a mean response time significantly longer than the ordinary transitive items. I argue that this difference is due to the difference in inferential distance: the ordinary transitive condition represents a causal inference, whereas the ordinary intransitive condition resembles a far-causal or temporal relationship. I thus argue that the results support the claim that ordinary intransitive verbs are semantically non-agentive or agentivity is not instantly evoked *via* pragmatics. On the other hand, AI intransitive items did not produce a mean response time significantly different from the AI transitive items. The lack of a significant difference in response times suggest the situation models constructed by the participants for the agent-implying intransitive items are similar to those for the AI transitive items in terms of inferential distance. Since the AI transitive items explicitly expressed direct causation, we may conclude that the AI intransitive items also involve causation. Thus, the findings of the priming experiment support the claim that the AI intransitive verbs are either semantically agentive (i.e., the semantic structure of these verbs involves an agent), or agentivity is instantly evoked *via* pragmatics. In other words, although the two types of verbs occur in the same syntactic structure, their processing differ.

These results have posted an interest question for the current theories of argument structure: If an agent is instantly evoked in the case of AI intransitive, can we attribute it to the semantics of the verbs, rather than to grammar or pragmatics?

Katz and Fodor (1963) argue that semantic knowledge is “any extragrammatical ability that a speaker can employ to understand the meaning of a sentence” (p. 174). They illustrate the bounds of semantics in two ways. First, if two sentences have the same structure but differ in meaning, then the difference in meaning must be handled by semantics. This is illustrated with the two sentences *The dog bit the man* and *The cat bit the woman*. Second, semantics is also responsible for cases where two sentences have different syntactic structures but identical in meaning. This can be illustrated with *The dog bit the man* and *The man was bitten by the dog*. Although the sentences are different, they are identical in meaning, and only those who have semantic knowledge of the language can tell that they are identical.

We can form similar sentences to illustrate the fact that the agentive properties is part of the meaning of the verb. First, its syntactic structure does not give it away that there is an implicit agent. In (22), both examples are in the form of an intransitive sentence, but only the first example is understood to have an external causer. This satisfies the first criterion.


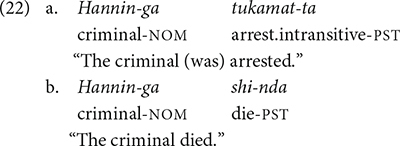


Second, the sentence in (22a) can be paraphrased into (23), which is a passive sentence. Although the syntactic structure is radically different, they are synonymous. This satisfies the second criteria. In other words, the agentivity issue that we are dealing with here is not a syntactic issue according to Katz and Fodor’s (1963) criteria.


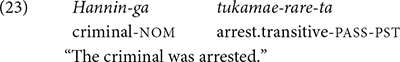


One might also ask whether the agentive interpretation is a result of semantics or pragmatics. Katz and Fodor (1963) argue that it is difficult to isolate the two:

“A limited (semantic) theory of how sociophysical setting determines the understanding of an utterance is possible but even such a theory blurs the distinction between the speaker’s knowledge of his language (his linguistic ability) and his knowledge of the world (his belief about matters of fact)” (p. 181).

One example that they used to illustrate their point is the differences in meaning among the sentences in (24). While the difference in meaning partly come from the different nouns in them (i.e., *junior, the lion*, and *the bus*), to correctly interpret these sentences, one will need to have knowledge about the world such as lions are often kept in cages whereas children and busses are not.


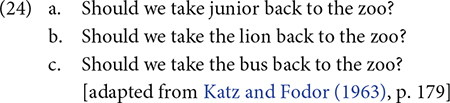


In my view, AI intransitive verbs present a very similar case. While one could argue that it is world knowledge that the act of arresting involves an external agent, one could also argue that it forms part of the meaning of the verb, precisely because the act of arresting must involve an agent. I leave this discussion open.

Another interesting question to ask is how different theories address the fact that agentivity is instantly evoked for one type of intransitive verbs and not the other. AI intransitive verbs have presented an interesting case for the lexicalist approach to argument realization, which in general holds that, if an agent exists in an event, it will be a core participant and will be realized as Subject. If agentivity is encoded as lexical information (e.g., in the event structure), one will need to explain why the agent, as a core participant, does not surface as the subject, as the ARC would predict. Rappaport Hovav and Levin (1998) claimed that some constant participants (e.g., “floor”) are optional because they are prototypical and recoverable for some verbs (e.g., *sweep*). One way to interpret the current findings in terms of this approach is to view the agent in AI intransitive verbs as an optional constant participant. However, this way of understanding raises more questions, such as under what conditions an agent is a structure participant and under what conditions it is a constant participant, as well as when it is optional. Recoverability could be related to other thematic roles in the event. For example, in the case of *tukamaru* “arrest,” if the theme is “the criminal,” the agent of arresting is likely to be recoverable. However, for a more neutral noun, such as “the actress,” the agent can vary based on different interpretations: it could be “the police,” or it could be “a hoard of paparazzi.” This discussion deserves future research.

In the syntactic approach where an external argument is added through syntax, it is an open question as to whether a VoiceP is added to the VP to provide an agent. One possibility is that an external argument has been added to VoiceP, thus leading a causative interpretation, but it is deleted in the surface form for some reason (e.g., because of lack of case).^6^ A discussion of how AI intransitive verbs differ from ordinary verbs in nature such that, for example, they fail to assign case, will be necessary.

From the perspective of Construction Grammar, the intransitive construction could be understood as one that is unique to Japanese. That is, the Japanese intransitive construction can encode causative events (unlike English). Goldberg (2010) claims that a word sense consists of two components: a profile, which is what the word asserts, and a background, which is what the word takes for granted. For example, the word *diameter* refers to a straight line that goes through the center of the circle. The line is the profile, but the sense of a diameter only makes sense when one presupposes the existence of a circle. Thus, the circle is the background. These two components together form the word sense of the word *diameter.* AI intransitive verbs can be understood in the same way. The intransitive verb *tukamaru* “arrest,” for example, profiles the patient and its change of state, and the background of the verb is the action that leads to this change of state of the patient. The use of agent-implying verbs in an intransitive construction thus forces the focus to fall on the patient. The Japanese intransitive construction is, therefore, similar to Goldberg’s (2005) Implicit Theme Construction, in that participant roles of an event do not necessarily appear as an argument. The difference is that instead of having an implicit theme, the Japanese intransitive construction has an implicit agent.

## Conclusion

This study has shown that AI Japanese intransitive verbs are processed differently from ordinary intransitive verbs in that the former instantly evokes a causative event whereas the latter does not. I argue that the findings of this study deserve consideration in the current theories of argument realization. Future research should address questions such as whether and how this information should be specified in the lexical entry or syntax, and what triggers pragmatic inferences in AI intransitive verbs but not in ordinary intransitive verbs.

## Data availability statement

The raw data supporting the conclusions of this article will be made available by the authors, without undue reservation.

## Ethics statement

The studies involving human participants were reviewed and approved by the Institutional Review Board, University of Pittsburgh and Human Research Ethics Committee, The Education University of Hong Kong. The patients/participants provided their written informed consent to participate in this study.

## Author contributions

ZL contributed to the design and implementation of the research, analysis of the results, and writing of the manuscript.

## Footnotes

1 While the term “intransitive” is sometimes used in the literature to refer to unergative verbs, in this article the term is simply used to distinguish verbs that take one argument only from those that take two arguments.

2 In Japanese, there are also transitive verbs that do not have an intransitive counterpart (e.g., *korosu* “kill”). These transitive verbs, however, are not the focus of this study.

3 There is a possibility that there is a penalty for reconstructing the situation since the agent is not expressed in the AI intransitive construction, resulting in longer response times. However, this will mean that we have even more reliable evidence to support the claim that AI intransitive verbs are semantically agentive if we find no significant difference between AI transitive and AI intransitive verbs. As reported in section “Results,” this was indeed the case.

4 These items involve *turu/tureru* “fish,” *tunagu/tunagaru* “connect,” *tutau/tutawaru* “transmit,” *hiyasu/hieru* “cool,” *kogasu/kogeru* “burn,” *nejiru/nejireru* “twist,” and *muku/mukeru* “peel.”

5 The value of eight was determined by the visual inspection of the boxplot of the data.

6 I thank the reviewer for pointing out this possibility.

## Supplementary material

Supplementary File Materials used in the norming task and the priming experiment.
