# Supplementary material for: The relationship between verb meaning and argument realization: What we learn from the processing of agent-implying intransitive verbs in Japanese
Source: Front Psychol. 2022 Aug 1;13:928649. doi: 10.3389/fpsyg.2022.928649 (PMC9377394; doi:10.3389/fpsyg.2022.928649)
Supplement: Supplementary File — Materials used in the norming task and the priming experiment. [file Data_Sheet_1.PDF]

Item	Verb		Transitive	Intranstiive	Question 1	Question 2	Statement in the norming task
1	**壊す／壊れる**	健二は家の中でバスケットボールをしていた。	バスケットをしている時、健二は時計を壊した。	バスケットをしている時、時計が壊れた。	バスケットボールで時計を壊すことができますか？	健二は家の中で遊んでいましたか？	時計がこわれたのは健二の責任だ。
2	**起こす／起きる**	恵子は家でピアノを弾いていた。	ピアノをひいている時、恵子は弟を起こした。	ピアノをひいている時、弟が起きた。	ピアノの音で人を起こすことができますか？	恵子はピアノを弾けましたか？	弟が起きたのは恵子が原因だ。
3	**鳴らす／鳴る**	健二は友達のうちの玄関まで来て、ドアの横のボタンを見つけた。	健二は少し待ち、そしてベルを鳴らした。	健二は少し待ち、そしてベルが鳴った。	ボタンでベルを鳴らすことができますか？	健二はいとこに会いに行きましたか？	ベルが鳴ったのは健二が原因だ。
4	**砕く／砕ける**	恵子は飲み物を冷やすのに、氷とアイスピックを持ってきた。	そして、恵子は氷を砕いた。	そして、氷が砕けた。	アイスピックで氷を砕くことができますか？	恵子はアイスピックを折りましたか？	氷が砕けたのは恵子がやったからだ。
5	**止める／止まる**	雅人は洗濯機に電源を入れたら、洗濯機から変な音がしているのに気づいた。	洗濯機を調べている時、雅人は洗濯機を止めた。	洗濯機を調べている時、洗濯機が止まった。	電源のボタンで洗濯機を止めることができますか？	洗濯機を直す必要がありますか？	洗濯機が止まったのは雅人が原因だ。
6	**冷やす／冷える**	洋子はパーティーの準備をしていて、飲み物を冷蔵庫に入れていた。	準備中に、洋子はビールを冷やした。	準備中に、ビールが冷えた。	冷蔵庫はビールを冷やすことができますか？	洋子はビールを冷蔵庫から取り出しましたか？	ビールが冷えたのは洋子のおかげだ。
7	**焦がす／焦げる**	ジョンは暖炉のそばでタバコを吸っていて、寝てしまった。	寝ている間に、ジョンはカーペットを焦がした。	寝ている間に、カーペットが焦げた。	タバコでカーペットを焦がすことができますか？	ジョンはズボンを焦がしましたか？	カーペットが焦げたのはジョンが原因だ。
8	**こぼす／こぼれる**	雅人はレストランでワインを注文しました。	ワインが来たが、すぐに雅人はワインをこぼした。	ワインが来たが、すぐにこぼれた。	ワインをこぼすにはグラスを触る必要がありますか？	雅人はレストランにいましたか？	ワインがこぼれたのは雅人が原因だ。
9	**動かす／動く**	洋子は家の外に車を見た。	洋子はその車に乗り、そして車を動かした。	洋子はその車に乗り、そして車が動いた。	車を動かすにはアクセルを踏む必要がありますか？	洋子はトラックを見ましたか？	車が動いたのは洋子がやったからだ。
10	**転がす／転がる**	雅人はゴルフクラブを使ってゴルフの練習をしていた。	雅人はボールに近づき、ボールを転がした。	雅人がボールに近づくと、ボールが転がった。	ゴルフをする時ゴルフクラブが必要ですか？	雅人はクラブを手に持っていましたか？	ボールが転がったのは雅人が原因だ。
11	**倒す／倒れる**	建築会社の社員はとても古い建物を壊すために、ダイナマイトを用意した。	彼らはそこに到着するとすぐに建物を倒した。	彼らがそこに到着するとすぐに建物が倒れた。	ダイナマイトで建物を倒すことができますか？	建物はとても古かったですか？	建物が倒れたのは建築会社の社員がやったからだ。
12	**回す／回る**	智也は自分の自転車を直そうとしていた。	自転車を持ち上げると、智也は自転車の車輪をくるくる回した。	自転車を持ち上げると、自転車の車輪がくるくる回った。	人の力で自転車の車輪をくるくる回すことができますか？	智也は自転車を持っていましたか？	自転車の車輪がくるくる回るのは智也がやったからだ。
13	**捻る／捻れる**	智也はペンチを持っていて、パソコンのケーブルを直していた。	直している時に、智也はワイヤーをねじった。	直している時に、ワイヤーがねじれた。	ペンチでワイヤーをねじることができますか？	智也は携帯を直していましたか？	ワイヤーがねじれたのは智也の責任だ。
14	**増やす／増える**	直人はテニスサークルの部長をしていて、新しい方針を提案した。	部長の任期中に、直人は部員の数を増やした。	部長の任期中に、部員の数が増えた。	部長の方針によって部員の数を増やすことができますか？	直人は学生ですか？	部員の数が増えたのは直人の成果だ。
15	**溶かす／溶ける**	裕子はチョコレートケーキを作ろうとしていて、チョコレートとお湯を取り出した。	ケーキを作っている時に、裕子はチョコレートを溶かした。	ケーキを作っている時に、チョコレートが溶けた。	お湯でチョコレートを溶かすことができますか？	裕子はフォークを使いましたか？	チョコレートが溶けたのは裕子がやったからだ。
16	**開ける／開く**	直人は家の前で鍵を探していた。	鍵を拾うと、直人はドアを開けた。	鍵を拾うと、ドアが開いた。	鍵でドアを開けることができますか？	直人は自分の鍵を持っていましたか？	ドアが開いたのは直人が原因だ。
17	**割る／割れる**	裕子はシャボン玉で遊んでいた。	裕子はシャボン玉を捕まえようとしている時に、シャボン玉を割った。	裕子はシャボン玉を捕まえようとしている時に、シャボン玉が割れた。	シャボン玉は触ると割れますか？	裕子はおもちゃの車で遊んでいましたか？	シャボン玉が割れたのは裕子が原因だ。
18	**剥く／剥けた**	京子は海に行って、日焼けした。	かゆくなって、京子は皮をむいた。	かゆくなって、皮がむけた。	手で皮をむくことができますか？	京子は海に行きましたか？	皮がむけたのは京子が原因だ。
19	**育てる／育つ**	京子は都内で一番古い高校に通っていた。	その頃、京子はひまわりを育てていて、そのひまわりが学校で一番大きいひまわりになった。	その頃、ひまわりが育っていて、そのひまわりが一番おおきいひまわりになった。	ひまわりを育てるには水をあげる必要がありますか？	京子は高校に通っていましたか？	ひまわりが育ったのは京子のおかげだ。
20	**傾ける／傾く**	直人は絵をながめていたら、地震が起きた。	地震が起きた後、直人は絵を左に傾けた。	地震が起きた後、絵が左に傾いた。	手で絵を傾けることができますか？	直人は絵を描いていましたか？	絵が傾いたのは直人のせいだ。
1	**壊す／壊れる**	雅人は庭でボールを蹴っていた。	ボールを蹴っている時、雅人は木の柵を壊した。	ボールを蹴っている時、木の柵が壊れた。	ボールで柵を壊すことができますか？	雅人は野球をやっていましたか？	柵が壊れたのは雅人のせいだ。
2	**起こす／起きる**	恵子は友達と電話で話していた。	電話で話している時、恵子は父を起こした。	電話で話している時、父が起きた。	電話の声で人を起こすことができますか？	恵子は電話で話していましたか？	父が起きたのは恵子が原因だ。
3	**鳴らす／鳴る**	雅人は親戚のおばさんの家に来て、インターホンを見た。	雅人は玄関に着いたとたん、インターホンを鳴らした。	雅人は玄関に着いたとたん、インターホンが鳴った。	インターホンを鳴らすにはボタンを押すことが必要ですか？	雅人は公園に行きましたか？	インターホンが鳴ったのは雅人が原因だ。
4	**砕く／砕ける**	直人はエアーガンで遊んでいた。	遊んでいる時、直人は電球をこなごなに砕いた。	遊んでいる時、電球がこなごなに砕けた。	エアーガンで電球をこなごなに砕くことができますか？	直人はエアーガンを手に持っていましたか？	電球がこなごなに砕けたのは直人のせいだ。
5	**止める／止まる**	恵子はケーキを作るため、ミキサーをかけた。	その後、恵子はミキサーを止めた。	その後、ミキサーが止まった。	ボタンでミキサーを止めることができますか？	恵子はケーキを食べていましたか？	ミキサーがとまったのは恵子の責任だ。
*6	**冷やす／冷える**	洋子はアイスコーヒーを作るのに、氷を取り出した。	そして、洋子はコーヒーを冷やした。	そして、コーヒーが冷えた。	氷でコーヒーを冷やすことができますか？	洋子はお茶を作っていましたか？	コーヒーが冷えたのは洋子が原因だ。
*7	**焦がす／焦げる**	洋子はコンロで魚を焼いていた。	焼いている時に、洋子は魚を焦がした。	焼いている時に、魚が焦げた。	コンロで魚を焦がすことができますか？	洋子は野菜を調理していましたか？	魚を焦がしたのは洋子のせいだ。
8	**こぼす／こぼれる**	直人はわんぱくな男の子だ。	直人は朝ごはんを食べている時、ミルクをこぼした。	直人は朝ごはんを食べている時、ミルクがこぼれた。	飲み物をこぼすには容器を触る必要がありますか？	直人は朝オレンジジュースを飲みましたか？	ミルクがこぼれたのは直人の責任だ。
9	**動かす／動く**	裕子はスーパーで買い物をしていた。	りんごを選ぼうとしているとき、裕子はカートを動かした。	りんごを選ぼうとしているとき、カートが動いた。	押すことによってカートを動かすことができますか？	裕子はりんごが欲しかったですか？	カートが動いたのは裕子が原因だ。
10	**転がす／転がる**	裕子は友達とサッカーをしていた。	遊んでいる時、裕子はボールを転がした。	遊んでいる時、ボールが転がった。	足でボールを転がすことができますか？	裕子は友達とサッカーをやっていましたか？	ボールが転がったのは裕子がやったからだ。
11	**倒す／倒れる**	智也は火事で壊れた家を修理しようとして、いろいろな工具を持ち出した。	家に入った時、智也は壁を倒した。	家に入った時、壁が倒れた。	ハンマーで壁を倒すことができますか？	その家は台風で壊れましたか？	天井が倒れたのは智也が原因だ。
12	**回す／回る**	智也はラジオの調子が悪いことに気がついた。	ラジオをいじっている時に、智也はアンテナを回した。	ラジオをいじっている時に、アンテナが回った。	アンテナを回すことによって受信が良くなりますか？	そのラジオにはアンテナがありましたか？	アンテナが回ったのは智也がやったからだ。
*13	**捻る／捻れる**	格闘技の試合で田中選手と鈴木選手が対戦した。	その時、田中選手は鈴木選手の手首をねじった。	その時、鈴木選手の手首がねじれた。	手で人の手首をねじることができますか？	鈴木選手は痛かったですか？	鈴木選手の手首がねじれたのは田中選手がやったからだ。
14	**増やす／増える**	京子はあまり食べないので、自分が痩せていると思っていた。	数ヶ月後、京子は体重を増やした。	数ヶ月後、京子は体重が増えた。	食べることによって体重を増やすことができますか？	京子は前より重くなりましたか？	体重を増やしたのは京子の努力だ。
15	**溶かす／溶ける**	京子はご飯を作っていて、冷蔵庫からバターを出した。	数分後、京子はバターを溶かした。	数分後、バターが溶けた。	調理でバターを溶かすことができますか？	京子はバターを使うつもりでしたか？	バターが溶けたのは京子が原因だ。
16	**開ける／開く**	健二は恵子に誕生日のプレゼントをあげた。	恵子はプレゼントをもらった時に、箱を開けた。	恵子はプレゼントをもらった時に、箱が開いた。	手で箱を開けることができますか？	恵子はプレゼントをあげましたか？	箱が開いたのは恵子が原因だ。
17	**割る／割れる**	小犬は風船を追いかけていた。	すると、小犬は風船を割った。	すると、風船が割れた。	爪や牙で風船を割ることができますか？	猫がその風船を割りましたか？	風船が割れたのは小犬が原因だ。
*18	**剥く／剥ける**	洋子は包丁でりんごを切ろうとした。	りんごを手にすると、洋子はりんごの皮をむいた。	りんごを手にすると、りんごの皮がむけた。	包丁で皮をむくことができますか？	洋子はりんごを食べようとしていましたか？	皮がむけたのは洋子が原因だ。
19	**育てる／育つ**	健二は草木の生えていない庭のある家に引っ越してきて、花の種を買ってきた。	数ヵ月後、健二は花を育てた。	数ヶ月後、花が育った。	肥料があると植物は良く育ちますか？	健二は種をもらいましたか？	花が育ったのは健二のおかげだ。
20	**傾ける／傾く**	健二は壁にある時計を写真にとろうとした。	その瞬間、健二は時計を傾けた。	その瞬間、時計が傾いた。	手で時計を傾けることができますか？	健二はカメラを持っていましたか？	時計が傾いたのは健二が原因だ。
1	**見つける／見つかる**	恵子は試験を受けていて、早く終わったので、答えを見直した。	答えを見直している時、恵子は間違いを見つけた。	答えを見直している時、間違いが見つかった。	間違いを見つけるのに注意が必要ですか？	恵子はご飯を食べていましたか？	間違いが見つかったのは恵子のおかげだ。
2	**捕まえる／捕まる**	警察は犯人を追っていた。	その後、警察が犯人を捕まえた。	その後、犯人が捕まった。	警察は犯人を逮捕しますか？	犯人は警察に連れに行かれましたか？	犯人が捕まったのは警察のおかげだ。
3	**助ける／助かる**	健二は子供が溺れているのを見た。	そして、健二は子供を助けた。	そして、子供が助かった。	大人は子供を助けることができますか？	子供は山に登りましたか？	子供が助かったのは健二のおかげだ。
*4	**釣る／釣れる**	直人は釣り竿を持って、湖に行った。	そして、直人は魚を釣った。	そして、魚が釣れた。	釣り竿で魚を釣ることができますか？	直人は湖に行きましたか？	魚がつれたのは直人のおかげだ。
5	**揃える／揃う**	洋子はケーキを作ることにしたので、スーパーに行った。	そして、洋子は材料をそろえた。	そして、材料がそろった。	材料をそろえるためには買い物をする必要がありますか？	洋子は小麦粉を買いましたか？	材料が揃ったのは洋子のおかげだ。
6	**定める／定まる**	直人は散弾銃を持って、山へ狩りに行った。	まもなく、直人は獲物の狙いを定めた。	まもなく、獲物の狙いが定まった。	獲物の狙いを定めるには散弾銃のスコープが要りますか？	直人は川に行きましたか？	獲物の狙いが定まったのは直人がやったからだ。
7	**備える／備わる**	洋子は新しい家に引っ越すことにして、家具屋さんに行った。	引っ越した後、洋子は家具を備えた。	引っ越した後、家具が備わった。	家具を備えるために買い物をする必要がありますか？	洋子はソファを買いましたか？	家具が備わったのは洋子のおかげだ。
*8	**つなぐ／つながる**	智也は馬を飼っていた。	馬の体を洗う時、智也はひもを柵につないだ。	馬の体を洗う時、ひもが柵につながった。	ひもを柵につなげる時結びますか？	智也は馬に餌をあげていましたか？	ひもが柵につながったのは智也がやったからだ。
9	**決める／決まる**	社員が集まって会議をした。	会議で、社員は新製品の発売日を決めた。	会議で、新製品の発売日が決まった。	発売日は話し合って決めるものですか？	新製品ができましたか？	発売日が決まったのは社員のおかげだ。
10	**混ぜる／混ざる**	裕子はケーキを作ることにして、砂糖や小麦粉やスプーンなどテーブルに置いた。	ケーキを作っている時、裕子は砂糖と小麦粉を混ぜた。	ケーキを作っている時、砂糖と小麦粉が混ざった。	スプーンで砂糖と小麦粉を混ぜることができますか？	裕子は塩を入れましたか？	砂糖と小麦粉が混ざったのは裕子がやったからだ。
11	**植える／植わる**	裕子は花が好きで、友達から花の種をもらった。	二ヵ月後、裕子は庭に花をたくさん植えた。	二ヵ月後、庭に花がたくさん植わった。	植物を育てるのに種をまくことが必要ですか？	裕子は木を育てましたか？	花がたくさん植わったのは裕子のおかげだ。
12	**散らかす／散らかる**	子供はおもちゃで遊んでいた。	遊んでいる時、子供はおもちゃを散らかした。	遊んでいる時、おもちゃが散らかった。	子供はおもちゃを投げたりしますか？	子供はおもちゃを片付けましたか？	おもちゃが散らかったのは子供のせいだ。
*13	**伝える／伝わる**	智也は光一に秘密を話した。	その後、光一は裕子に秘密を伝えた。	その後、裕子に秘密が伝わった。	秘密を伝えるには言葉が必要ですか？	光一は智也から秘密を聞きましたか？	裕子が秘密を知ったのは光一のせいだ。
14	**届ける／届く**	雅人は郵便局に行って、恵子に荷物を送った。	三日後、郵便局が荷物を届けた。	三日後、荷物が届いた。	郵便局は荷物を配達しますか？	恵子は雅人から荷物をもらいましたか	荷物が届いたのは郵便局のおかげだ。
15	**埋まる／埋める**	雅人はシャベルを使って、宝を隠そうとしていた。	そして、雅人は庭に宝を埋めた。	そして、庭に宝が埋まった。	物を埋めるにはシャベルで穴を掘りますか？	雅人は宝を盗まれましたか？	宝が埋まったのは雅人がやったからだ。
16	**染める／染まる**	川の隣に工場ができた。	その後、工場（の汚水？）が川を黒く染めた。	その後、川が黒く染まった。	汚水で川が黒くなりますか？	川は前よりきれいになりましたか？	川が黒くなったのは工場の責任だ。
17	**はめる／はまる**	京子は友達と指輪を買いに行った。	試そうとして、京子は指輪をはめた。	試そうとして、指輪がはまった。	手で指輪をはめますか？	京子は指輪が欲しかったですか？	指輪がはまったのは京子がやったからだ。
18	**つける／つく**	健二は真っ暗な部屋にいて、スイッチを探していた。	そして、健二は電気をつけた。	そして、電気がついた。	スイッチで電気をつけることができますか？	健二は部屋にいましたか？	電気がついたのは健二の責任だ。
19	**抜く／抜ける**	京子は歯医者さんに行った。	その後、歯医者さんが歯を抜いた。	その後、歯が抜けた。	抜歯用ペンチで歯を抜けることができますか？	京子は図書館に行きましたか？	歯が抜けたのは歯医者さんがやったからだ。
20	**建てる／建つ**	ある会社が土地を買い、建築会社にホテル建設を依頼した。	その後、建築会社がホテルを建てた。	その後、ホテルが建った。	ビルを建てるには人手がたくさん要りますか？	**会社は土地を買いましたか？**	ホテルが建てたのは建築会社がやったからだ。

*Items that were removed from analysis.
